# Patient Adherence and Persistence with Imatinib, Nilotinib, Dasatinib in Clinical Practice

**DOI:** 10.1371/journal.pone.0056813

**Published:** 2013-02-20

**Authors:** Fiorenzo Santoleri, Paola Sorice, Ruggero Lasala, Rosa Carmela Rizzo, Alberto Costantini

**Affiliations:** Hospital Pharmacist, Hospital Pharmacy, General Hospital of Pescara, Pescara, Italy; B.C. Cancer Agency, Canada

## Abstract

**Introduction:**

The aim of this study is to evaluate adherence and persistence of patients treated with Imatinib, Nilotinib or Dasatinib, also giving economic evaluations on therapy costs for Received Daily Dose (RDD).

**Materials and Methods:**

In this retrospective study, we took into account 3 years from 1st Jan. 2009 to 31st March.2012. Treatment adherence was quantified utilizing ratio between RDD and PDD (Prescribed Daily Dose). Persistence is reckoned taking into account the actual therapy days, comparing posology with supplied dose, drawing the graph using Kaplan-Meir method.

**Results:**

Adherence results in values between 0.8 and 1.0 for Nilotinib (Adh  = 0.93), Imatinib (Adh  = 0.83) and Dasatinib (0.85). Imatinib has better persistence, 90% of patients in treatment exceed one year of treatment versus 83.3% for Nilotinib and 80% for Dasatinib. The cost per single day of treatment (cost per RDD) was € 39.41 for Imatinib, € 113.60 for Nilotinib and € 94.84 for Dasatinib.

**Conclusion:**

Patients with CML have a loose of adherence both in first line with Imatinib and in second line of therapy with Dasatinib and Nilotinib. Loss of adherence remains a big problem and could be minimized by a patient-oriented project invlolving physicians, nurses, pharmacists and caregiver.

## Introduction

Over the past decade self administration of oral chemotherapy has increased because of the availability of novel therapeutic agents. Oral therapy gives numerous advantages to the patients, such as potential increase in the quality of life and potential reduction in travel costs and use of health care resources. [1], [2] Unfortunately patients often have no sufficient education and information about the use of oral chemotherapeutic. [3] Whereas administration in hospital, where the prescribed medication, dose, regimen and response to therapy are monitored by physicians, pharmacists and nurses, at home, patients or caregivers are susceptible of errors, nonadherence and increased adverse events as a result of a lack of coordinate care. Errors in oral administration can include incorrect dosing and limited monitoring with underdosing or overdosing, with possible consequences of serious toxicity, morbidity and mortality. [4], [5] Patient adherence and persistence to oral anticancer drugs are defined as an emerging issue in modern oncology, representing a new challenge that has as its aim patients’ safety and effectiveness of treatment. [6] As for Imatinib for example, it is demonstrated that adherence is a critical factor to achieve molecular responses in patients with Chronic Myeloid Leukemia (CML). [7] Adherence is correlated to the good outcome of treatment; great attention is now given to adherence to therapy in the interest of patients. So adherence becomes an important factor in patients’ clinical history, resulting essential in clinical response to pathology. If there is a good compliance of patient and adherence to treatment, therapy has less probability to fail. In literature several trials designed to improve medication and adherence are reported. The use of calendar packaging [8], [9], [10], has proved to improve adherence to self-administration. There are several methods to calculate Adherence. In fact in literature there isn’t one single usual method given common currency. MPR (Medication Possession Ratio), CMA (Continuous Measure of Adherence), number of days of medication supplied within the refill, PDC (Proportion of Days Covered) are mostly used. [11] The role of pharmacist is important to underline duplicate therapies, drug interactions, side effects, lack of efficacy and untreated condition. [12] The aim of this study was to monitor adherence and persistence to oral chemotherapy with the aid of a single patient-oriented application program, taking into account drug used parameters as Received Daily Dose (RDD) and Prescribed Daily Dose (PDD). For this study, hospital pharmacists developed a pharmaceutical database built up ad hoc for the calculation of adherence, named PharmaDDSS, in which pharmacist who dispenses drug inserted useful data to calculate that parameter. The study includes three oral chemotherapeutic agents used for CML: Imatinib, Nilotinib and Dasatinib, suggesting a new method to evaluate adherence, as this parameter is of great bearing in successfulness of treatment, and of persistence with drug until patient’s death or progression of pathology or toxicity of therapy.

### DDD

Defined Daily Dose (DDD) is the assumed average maintenance dose per day for a drug used for its main indication in adults. [13] It is used on worldwide scale as International standard for drug utilization studies; for antineoplastic agents no DDDs have been established because of highly individualized use and wide dosage ranges. Dose used differs substantially on the ground of various types and severity of neoplastic diseases, and on the basis of large use of drugs in combination therapy. DDDs provide a fixed unit of measurement indipendent from price, currency, package size and strength enabling the researcher to assess trends in drug consumption and to perform comparison between population groups. DDD represents only a rough estimate of consumption of drugs and not an exact status of utlization in real clinical practice. Dose can change considerably depending on association, weight of patient, pathology, and those variables are not considered in the definition of DDD.

### PDD

PDD, the prescribed daily dose, is an indicator of intention to treat. [13] That piece of information is important to have at hand a faithful report of the real prescriptive tendency with reference to a given drug. In fact the prescribed dose can change depending on the patient’s response to therapy. So PDD is very important, mainly with drugs, such as the ones in study, for which it can be different from the Defined Daily Dose (DDD). For example, Dasatinib is available in four dosages: 50, 80, 100 and 140 mg, so it would be difficult to assign a single DDD. On the contrary, a dosage reduction is possible for Imatinib, so the PDD changes from 400 to 200 mg. DDD is the standard unit of measurement, defined as “daily maintenance dose of a drug used for its main purpose in adult”. Although it is universally accepted as the standard quantity measurement index, it shows several limits when we try to investigate the actual use of a drug. In this study, PDD has been extrapolated from the prescribed treatment and updated at every renewal. In this way, a PDD has been calculated for each patient, depending on the doctor’s prescription and on the treatment duration. PDD values presented for each drug and for each dose have been reckoned as the average of all prescribed doses.

### RDD

Received Daily Dose (RDD) can be defined as the weighted average daily dose received by patient weighted on the period of interval between refills. RDD is calculated as ratio between the total doses received by patient and treatment days. Treatment days are calculated as the interval between two next dispensing of the same drug to the same patient. It's possible to calculate it because of a specific database built ad hoc. With this method the dose dispensed to the patient from the hospital pharmacist is also considered the self-administered at home. Problems with this parameter are linked with real administration of drug in timing and dosage, when the patient returns to hospital pharmacy before or after the due date. This gap is limited by statistical extrapolation calculated on each patient. In this study, compared to a given patient, RDD is calculated by dividing the total amount of active ingredient during the reference period by the days of that period. We resort to weighted mean because intervals between each refill are different, while we want to reckon an exact value for the real use of the drug for each single patient, for each treatmenet day (i.e. a shorter interval, e.g. 5 days, cannot be regarded as a longer one of 40 days, they do have a different weight on the final result). It is very simple to reckon weighted mean using an Excel program, using sumproduct function.
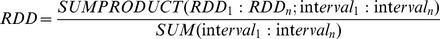


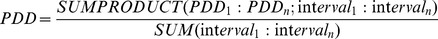



### DDD, PDD, RDD, Adherence, Persistence

Adherence is the extent to which a patient acts in accordance with the Prescribed dosing regimen. The unit of measure is administered doses per defined period of time, reported as the proportion (%) of prescribed doses taken in the prescribed time interval, as measured by the period of time [13], [14], [15], [16], [17] DDD, PDD and RDD can be used to estimate patient’s adherence to treatment (RDD/PDD), the respect of the standard dose by the physician (PDD/DDD) and appropriateness of treatment (RDD/DDD). In this observational study, medication adherence was calculated as ratio between RDD to PDD. In this way adherence is not a value between 0 and 1, but it can be 0 when patient doesn’t take the drug, >0 and <1 when patient take less drug than prescribed by physician, 1 if Prescribed dose and Received dose are perfectly equivalent, bigger than 1 if the received dose is higher than prescribed dose.



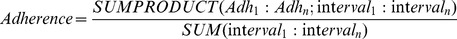



Persistence with therapy is defined as the accumulation of time from initiation to discontinuation of therapy, measured by time metric. [18] So we can describe persistence as the duration in time of therapy with the same drug or we can consider persistence as the continuation of therapy beyond a fixed time and in this case it will corresponde to dichotomous variable yes/no, that’s the Estimated Level of Persistence (ELPT model). In this study persistence is calculated in both ways listed, quantifying not only the total duration of therapy, but also the intensity of medication-taking within this interval. [19], [20] Persistence is an important factor to evaluate the response to treatment. In fact therapy is suspended in case of progression of pathology, death of patient or toxicity. We calculate the drug persistence as the effective days of therapy and in basis of Prescribed Daily Dose, we evaluate if patients have more or they suspend therapy before one year of treatment.

## Materials and Methods

This observational study was carried out in the 2012 in the Hospital Pharmacy of Pescara (Italy). The study design was approved by the hospital ethics committee of Pescara. The written consents were not given by the patients for their information because this is a observational retrospective study as regulated by the Italian Agency of the drug with the “Guidelines for the classification and management of observational studies on drugs. As described in the guidelines available on the website “agenziafarmaco.gov.it”. In the case of studies that do not involve a direct relationship with the patient, it is not necessary to administer the privacy of the patient and the informed consent form. The analyzed data were already in the hospital pharmacy database used daily for the clinical practice. All data were analyzed anonymously. Each patient was identified with a personal number. Patients were aware that their data were stored in a specific database, but were not informed that these data were used for research purposes. This procedure has been disclosed to the Ethics Committee that, in accordance with national legislation, approved it. Every patient in therapy with Imatinib, Dasatinib and Nilotinib for Chronic Myeloid Leukemia was enrolled in study, in the period of time from 1^st^ January 2009 to 31^st^ March 2012. The data of prescription and consumption of oral chemotherapy were recorded in a database built specifically to follow the patient throughout the care pathway. In this database, named PharmaDDSS, the following data were recorded by the hospital pharmacist: patient demographics, drug used and its indication as prescribed by the physician, Defined Daily Dose (when present) (DDD), Prescribed Daily Dose (PDD) by physician and Received Daily Dose (RDD) by pharmacist. The first three parameters were collected by the hospital pharmacist through consultation with the treatment plan, in which that records have to be filled in by the physician, together with the references related to prescribed drug, dosage, estimated duration of validity of the plan. The hospital pharmacist records all this data in PharmaDDSS. The DDD is the assumed average maintenance dose per day for a drug used for its main indication in adults. Although DDD represents an important drug consumption indicator, it does not show the recommended or prescribed dose, not even the one actually taken by the patient based on the physician's prescription, which differs because of association with other drugs, patient's general health (sometimes dosage must be reduced because of side effects), patient's weight and age. Each patient has in his medical record the daily dose as indicated by the physician and the dose of drug received in refill. During the year this dose may be changed by the physician and then updated by hospital pharmacist. In this way, each patient has a personal record where all doses and change of doses are recorded. The daily dose prescribed is PDD, while RDD is calculated by dividing the dose received by the patient for treatment days. Treatment days are considered as difference between the first and second date of dispensation of drug by the pharmacist in hospital pharmacy. In this way, the hospital pharmacist follows the patient during his health treatment, thus getting to know the medication adherence calculated as ratio between RDD and PDD. RDD, in fact, can be defined as the dose really taken by the patient and PDD represents the intention to treat. The optimum of medication adherence is near to 1.



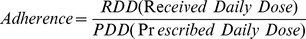



**Adherence  = 0→** Patient hasn’t taken drug, that condition is not possible if patient returns in pharmacy for second refill;

**0< Adherence<1→** Patient takes less drug then prescribed;

**Adherence ≈ 1→** Received dose and prescribed dose are equivalent, optimum of Adherence;

**Adherence>1 →** Patient has taken more drug than prescribed.

Differently from DDD, with PDD we consider all the therapeutic indications for which the drug is used, considering all patients who take that drug.

Drug persistence with therapy was calculated as total days of treatment with the same drug for each patient; thanks to PharmaDDSS program, used for calculation of adherence, but useful for persistence too, the hospital pharmacist records data about the dispensed dose, the date of dispensation, the prescribed dose. So we can calculate the persistence as the total days covered by therapy; we estimate the total days of treatment adding all the intervals between refills of drug and adding also the days covered by the last refill, obtained dividing the last dose refilled in hospital pharmacy by the last prescribed dose. For Persistence, we consider the effective days in which patient has taken drug, deducting from persistence NPD (Non Persistence Days) on the basis of Prescribed Dose. Comparing the persistence with three different drugs (Imatinib, Nilotinib, Dasatinib), we considered only the common therapeutic indication, the LMC Ph+, excluding for Imatinib the Gastrointestinal Stromal Tumor and hypereosinophilic syndrome.



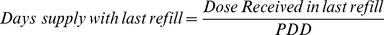



Interval 1 =  time in days that elapses between first and second refill
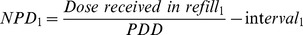


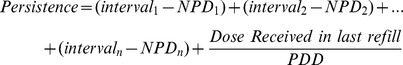



Persistence as calculated here considers only effective days of therapy based on the prescribed dose, and not the mere interval between first and last administration of drug. In fact we deduct the days of non-persistence from total persistence, and in this case we can correlate adherence with persistence with therapy multiplying the sum of total days by the value of adherence.
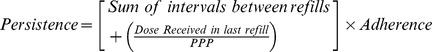



This innovative method of calculation of Adherence and Persistence doesn’t consider DDD, but PDD. In this way it overcomes the usual methods accepted by scientific society, like MPR, PDC (all based on DDD). With this study we introduce a new fundamental parameter in literature, the PDD, indispensable to calculate both adherence and persistence.

## Results

**Figure 1:**
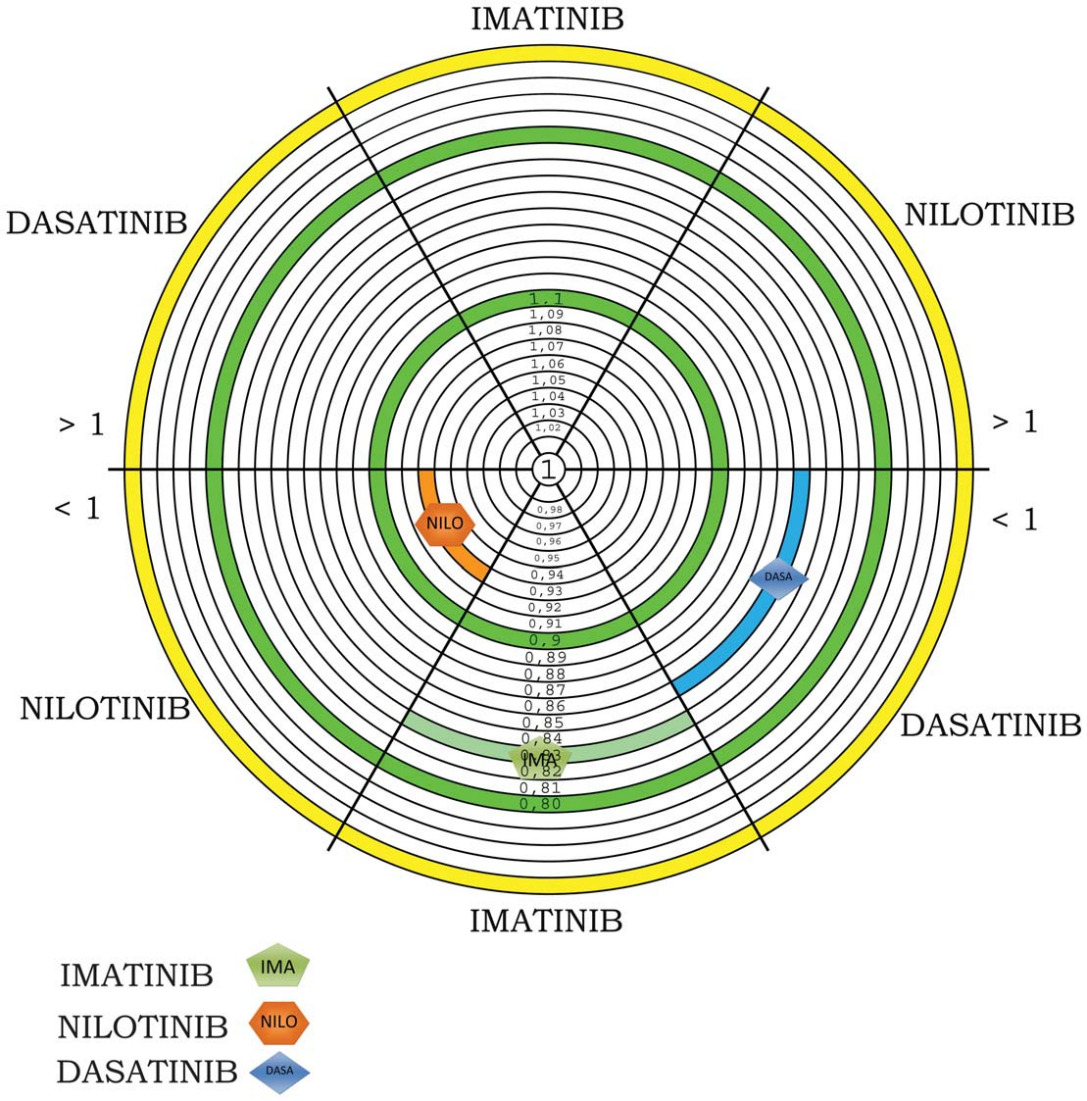
Medication adherence for Imatinib, Nilotinib and Dasatinib.

**Figure 2:**
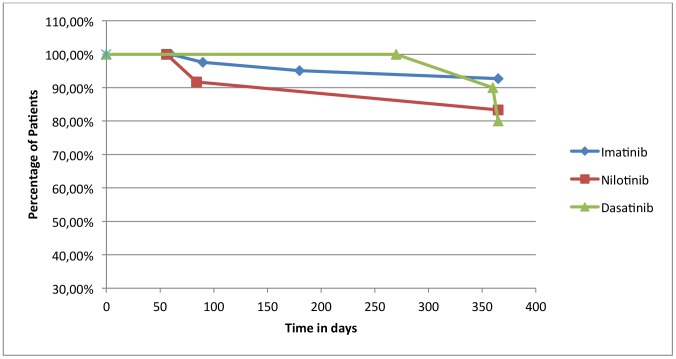
Kaplan Meier Representation of Persistence to one year of therapy with Imatinib, Nilotinib and Dasatinib.

**Figure 3:**
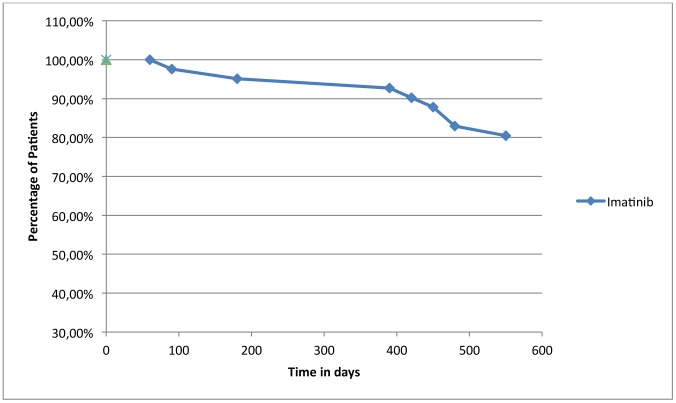
Kaplan Meier Representation of Persistence to 18 months of therapy with Imatinib.

The observational study was carried out from 1^st^ January 2009 to 31^st^March 2012. The number of patients recorded was 91 for adherence, 66 for persistence. Number of patients, sex, age, and days of treatment (TDT), PDD and RDD were collected in Table 1 divided for oral targeted agent. All patients included in the study are in chronic phase of CML. All patients in therapy with Imatinib are always in first line of treatment, while all patients in treatment with Nilotinib and Dasatinib have been previously treated with Imatinib, showing intolerance or resistence; three of thirteen patients in treatment with Dasatinib have previously taken both Imatinib and Nilotinib. In addition, none of the patients included in the study belongs to clinical trials, in fact one of the objectives of the present study is to follow the patient in the management of therapy trying to describe the quality of treatment in daily practice, the behaviour of patients in daily clinical practice, without the support of health personnel. Three hematologists, all belonging to the same department, followed all patients in study. In the analysis of medication adherence there are not significant differences between patients followed by different physicians. For each oral cancer drug dispensed in the hospital pharmacy of Pescara there was medication adherence (ADH) (Fig. 1). Values of adherence were 0.93 for Nilotinib, 0.85 for Dasatinib and 0.83 for Imatinib with a loss of adherence of 7%, 15% and 17% respectively. The RDD of Imatinib correspondes to 321.33 mg, while the PDD is 383.19 mg with an adherence equal to 0.83; the RDD for Dasatinib correspondes to 86.22 mg while the Prescribed Daily Dose is 95.54 mg with an adherence equal to 0.85; the RDD for Nilotinib correspondes to 632.54 mg while the PDD is 687.05 mg with an adherence equal to 0.93. PDD is calculated on the basis of physician’s prescriptions; for Imatinib the doses of prescriptions is 400 mg/day in all cases except seven patients who take 300 mg/day, 1 patient 200 mg/day and 1 patient 600 mg/day; for Nilotinib dosage adjustament involves 5 patients, who take 400 mg/day instead 800 mg/day (this study dates back the introduction of formulation of Nilotinib 150 mg); for Dasatinib instead we have that 3 patients take 50 mg/day, 2 patients 140 mg/day, the other patients take 100 mg/day. To calculate persistence, we consider all the patients in treatment with Imatinib, Nilotinib and Dasatinib, enrolled to therapy before 31st March 2011, so that we have in study only patients who have the possibility to make at least one year of therapy. In fact we extrapolated the data related to patients till 31st March 2012. Then to calculate persistence with Imatinib we enrolled in study 42 patients, for persistence with Nilotinib 12 patients and for persistence with Dasatinib 10 patients. Imatinib has better persistence, 90% of patients in treatment exceed one year of therapy, while 83.3% of patients in treatment with Nilotinib exceed to one year of therapy, 80% of patients in treatment with Dasatinib has exceeded one year of therapy. (Fig. 2) Persistence rate was represented with Kaplan Meier survival analysis and resulted identical among treatments with Imatinib, Nilotinib and Dasatinib, using Log Rank Test. Persistence with drugs to one year and half is possible only for Imatinib. For Nilotinib and Dasatinib the population in study was very reduced because of more recent commercialization. To analyze persistence with Imatinib to 18 months of therapy we have considered all the patients enrolled to treatment with drug before 30thSeptember 2010. In total we have a population of 41 patients. (Fig. 3). The cost per single day of treatment (cost per RDD) was € 39.41 for Imatinib, € 113.60 for Nilotinib and € 94.84 for Dasatinib.

**Table 1:** Number of patients, sex, age and drug used parameters for Imatinib, Nilotinib, Dasatinib.

	Imatinib	Nilotinib	Dasatinib
Number patients	63	15	13
Sex:			
Male	21	6	7
Female	42	6	9
Age			
Median	62	52	51
Range	14–88	34–82	27–77
Line of therapy			
First line	63	–	–
Second line	–	15	10
Third line	–	–	3
RDD Weighted Average ± SD	321.33±97.04	632.54±154.45	86.22±27.1
PDD Weighted Average ± SD	383.19±40.04	687.05±175.41	95.54±27.1
Persistence with thearpy			
Range	60–365	56–365	270–365
Median	365	365	365

## Discussion

The presented data highlights the safety of the drugs used for chronic myeloid leukemia and that the side effects and adverse events are not so severe as to affect adherence to therapy. The different dosage regimens of the 3 drugs, Imatinib 4 tablets once a day, Nilotinib two tablets twice a day and Dasatinib one tablet once a day, appear to affect minimally adherence to the treatment, which could be the cause of the lower adherence of Imatinib; but does not explain the very similar adherence of Dasatinib. This data suggest that the adherence is not influenced by therapeutic regimen, two tablets twice a day of Nilotinib versus one tablet once a day of Dasatinib. These evidences are reported in literature in a retrospective analisys between Nilotinib and Dasatinib. [21] The adherence of Dasatinib (0.85) probably depends on the fact that approximately 25% of patients has taken it after failure of two prior therapies (Imatinib and Nilotinib); in this case confidence in therapy has a fundamental role in the adherence to therapy and then in the good treatment outcome. Therefore, two factors seem to be important to improve adherence to treatment: absence of significant side effects and active involvement of the patient who becomes the active part of his course of treatment. The most important side effects were leukopenia and neutropenia which could be cause of loss of adherence, because the phisician might decide to interrupt the treatment until values become normal again. Gastrointestinal secondary side effects may also occur, but, generally, are well tolerated by patients who continue to take drugs. The persistence rate is very similar for the three drugs, despite the fact that chronic phase CML patients in this study are at different stages of their disease history. In fact Imatinib is used in first line and Nilotinib and Dasatinib for second line, after failure of Imatinib. This study highlights a new method of calculation of consumption and utilization of drugs. In the specific case we consider targeted therapies, which haven’t as yet a standardized International dosage like DDD. DDD is a temperametal value of consumption of drug in clinical reality. It gives only an indicative data, a mere statistical measure based on principal therapeuthic indication, for the standard weight and age of patients, and doesn’t give a real information about the real utilization in clinical practice, in every single case in which the drug is used, as we do in this study.

### Conclusions

Investigating the adherence to treatment is important in order to know how respect of posology is linked with efficacy of treatment. In fact, patient’s adherence to therapy in the first year is essential to treat well the CML. Adherence to treatment can be correlated to good persistence to therapy with the drugs. In fact many studies in literature and real practice correlate adherence to efficacy of therapy and in this specific case with CCyR (Complete Cytogenetic Response) that results proportional to adherence to therapy. Nowadays it is demonstrated that the benefit of therapy is strictly correlated to day-to-day administration of therapy. In this way it is important to try to follow the patient in his care pathway, from diagnosis to drug administration. The good management of therapy is strictly correlated to a complete patient involvement. The medication-adherence’s studies are a first step to understand the extent of the problem. For this reason we regard it as very important to develop an economic and accurate method to calculate adherence, based on the PDD and RDD, with the utilization of a database developed ad hoc. The better economic profile of Imatinib compared to Dasatinib and Nilotinib, calculated as cost per RDD, supports the importance to choose it for the first time in the treatment of CML. Loss of adherence remains a big problem and could be minimized by a patient-oriented project involving physicians, nurses, pharmacists and caregivers.
